# Early diagnosis of ataxia telangiectasia in the neonatal phase: a parents’ perspective

**DOI:** 10.1007/s00431-019-03479-5

**Published:** 2019-11-11

**Authors:** M. H. D. Schoenaker, M. Blom, M. C. de Vries, C. M. R. Weemaes, M. van der Burg, M. A. A. P. Willemsen

**Affiliations:** 1 Department of Pediatric Neurology, Amalia Children’s Hospital, Donders Institute for Brain, Cognition and Behavior, Radboud University Medical Center, Geert Grooteplein Zuid 10, 6525 GA Nijmegen, The Netherlands; 2 Department of Pediatrics, Laboratory for Immunology, Leiden University Medical Center, Leiden, The Netherlands; 3 Department of Medical Ethics and Health Law, Leiden University Medical Center, Leiden, The Netherlands; 4 Department of Pediatrics, Amalia Children’s Hospital, Radboud University Medical Center, Nijmegen, The Netherlands

**Keywords:** Ataxia telangiectasia, Early diagnosis, Parents perspective, Parents opinion

## Abstract

Ataxia telangiectasia (A-T) is a severe neurodegenerative disorder with variable immunodeficiency. Together with the Dutch A-T community, we investigated the opinion of A-T parents on an early A-T diagnosis in the asymptomatic phase of the disease. During an annual national meeting for A-T patients and families, the topic of an early A-T diagnosis was discussed in relation to the recent introduction of neonatal screening for severe combined immunodeficiency (SCID) in the Netherlands. Based on the discussion, individual arguments were identified and processed into a questionnaire, which was sent out to 64 A-T parents (32 families). Arguments included were insecurity to diagnosis, possible medical advantages, appropriate genetic counseling and family planning, loss of “golden” year(s), and early cancer screening for parents. The response rate was 55% (*n* = 35 parents). Twenty-six (74%) parents felt that the advantages of an early diagnosis outweighed the disadvantages, five parents thought that the disadvantages would outweigh the advantages (14%), and four parents did not indicate a preference.

*Conclusion*: The majority of parents of a child with A-T would have preferred an early diagnosis during the asymptomatic phase of the disease, because the uncertainty during the diagnostic process had had a major impact on their lives. In addition, the knowledge of being carriers of an *ATM* gene mutation influenced decisions about family planning. Parents who opposed against an early diagnosis emphasized the joy of having a seemingly healthy child until diagnosis.

**Table:** 

**What is Known:** *• Ataxia telangiectasia (A-T) is a devastating DNA repair disorder with a huge impact on quality of life of patients and their parents.* *• Patients with A-T may incidentally be identified at birth as the consequence of neonatal screening for severe combined immunodeficiency (SCID).*	
**What is New:** *• The majority of Dutch parents of A-T patients (74%) would have preferred an early diagnosis of their child in the asymptomatic phase of the disease.* *• Major arguments for an early A-T diagnosis were (1) the experienced insecurity in diagnostic trajectories and its impact on families and (2) the knowledge of being ATM mutation carriers when deciding about family planning. An argument against an early diagnosis is losing the joy of having a seemingly healthy child until diagnosis.*

**Electronic supplementary material:**

The online version of this article (10.1007/s00431-019-03479-5) contains supplementary material, which is available to authorized users.

## Introduction

Ataxia telangiectasia (A-T) is a devastating, autosomal recessively inherited disease with a huge impact on quality of life of patients and their parents. A-T is a DNA repair disorder, caused by mutations in the *Ataxia Telangiectasia Mutated* (*ATM*) gene, leading to neurodegeneration with progressive ataxia, telangiectasias, predisposition to malignancies, sensitivity to radiation, and immunodeficiency [17]. Patients with classic A-T have no symptoms in the first year of life; progressive symptoms, however, start shortly thereafter. An early diagnosis helps to start up A-T specialized care regarding the medical support for pulmonary function, prophylactic antibiotics or immunoglobulins for recurrent infections, and an adapted treatment for malignancies [17, 18]. Unfortunately, a curative treatment for A-T is not yet available, and most patients with the classic form of the disease die before the age of 30 years.

During the past years, newborn screening (NBS) for severe combined immunodeficiency (SCID) has become available and has been introduced in several countries [11]. Newborns with SCID face the risk of life-threatening infections, caused by very low numbers of T cells. Early diagnosis is essential, as SCID patients treated with stem cell transplantation before the age of 3.5 months or before infections have occurred have a significantly improved survival compared with those transplanted later or when infectious complications have accumulated [9, 15]. NBS for SCID is based on quantification of T cell receptor excision circles (TRECs) in dried blood spots (Guthrie cards) of newborns. TRECs are formed during development of T cells and are used as biomarker for the presence of naive T cells. SCID patients do not have (functional) T cells and therefore lack TRECs [6]. Low/absent TRECs can also be identified in neonates with T cell impairment syndromes, newborns with T cell impairment secondary to other neonatal conditions, or patients with idiopathic lymphocytopenia [1, 2, 13, 19]. Flow cytometry and genetic analysis are therefore required as confirmatory diagnostics to distinguish true SCID patients from these incidental findings.

It is known that part of the newborns with A-T have low TREC levels, and therefore, some (at that stage pre-symptomatic) A-T patients may be identified incidentally during NBS for SCID [5, 14]. In April 2018, an implementation pilot study for NBS for SCID started in the Netherlands (SCID-screening Research in the Netherlands with TRECs (SONNET-study), www.sonnetstudie.nl) [4]. In the Netherlands, screening for treatable disorders is undisputed; however, the discussion continues about screening for non-treatable disorders. Current guidelines of the Health Council of the Netherlands advise not to screen for non-treatable disorders and not to report (incidental) findings that may refer to untreatable disorders. However, when there is potential health gain or prevention of health loss for a child with an early diagnosis, this discussion can be re-opened in case there is supporting scientific evidence [8]. The fact that A-T should be regarded as untreatable potentially leads to the unwanted situation of identifying a patient with A-T at a pre-symptomatic stage, based on NBS for SCID. From the experience of our national reference center for A-T at the Radboud University Medical Center, we know that the diagnostic process for A-T may take a long time and may include many procedures, e.g., lumbar punctures, muscle biopsies, and diagnostic x-rays (which are potentially harmful in the context of a DNA-repair disorder). Family members with a single *ATM* mutation have an increased risk for developing cancer (especially breast cancer in women) and cardiovascular diseases [10, 16, 20]. All the facets around the diagnosis of A-T combined, including the psychological stress, make it an interesting question whether parents of A-T patients would favor the possibility of an early diagnosis of A-T directly after birth, as a consequence (in fact an incidental finding) of SCID screening. In this study, a questionnaire was developed to investigate whether parents of a Dutch cohort of A-T patients would consider an early diagnosis beneficial or whether they would consider it harmful (taking away “the golden year(s),” i.e., the happy time before onset of symptoms). As NBS for SCID is introduced in many other countries, this research could contribute to the discussion whether A-T (or other untreatable disorders) should be diagnosed at a very early age when possible [12].

## Methods

Once every one or two years in the Netherlands, a national meeting with all A-T families is organized to give an update of recent developments in our clinic and in science, to discuss the progress in the medical literature, and simply to meet each other. During one of these meetings, parents and professionals discussed whether an early diagnosis of A-T would be advantageous. Based on this discussion, individual arguments were identified and processed into a questionnaire. Potential arguments were uncertainty up to the diagnosis, possible medical advantages, genetic counseling and family planning including potential prenatal diagnostics, loss of happy years, and early cancer screening for parents. To test the questionnaire, all doctors, nurses, and paramedics involved in the A-T team were sent a questionnaire. After this, the questionnaire was improved and sent to all Dutch parents of an A-T patient. Every household received two questionnaires (one for each parent). For every statement, a five-scale option was provided: strongly agree, agree, neutral, disagree, and strongly disagree. For the final question, three options were given: “the advantages outweigh the disadvantages,” “the disadvantages outweigh the advantages,” or “I don’t know.” Parents were given the opportunity to motivate their definitive choice in an open box. The study was approved by the local medical ethical committee (METC 2018-4518).

## Results

In total, 64 A-T parents (32 families) received a questionnaire. The response rate was 55% as 35 A-T parents filled in the questionnaire. When parents filled in the questionnaire together, the questionnaire was counted twice. One grandmother filled in a questionnaire (instead of father); these data were included in the results. Fifteen A-T children had parents who both filled in a questionnaire, and five children had one parent who filled in the questionnaire. The cohort which replied to the questionnaire consisted of 21 classic A-T and 1 variant A-T (44 years old). The average age of alive classic A-T patients is 11 years (range 2–30), and five classic A-T patients deceased at an average age of 20 (range 14–26, 1 had missing data). The average age at diagnosis of A-T was 4.9 years old (range 1–10 years) for classic A-T. One variant A-T was diagnosed around 32 years old. No differences were observed between subgroups in this small and heterogeneous group of respondents.

### Time to diagnosis

The first statement was aimed to verify whether parents would like to have known the diagnosis A-T shortly after birth in their specific situation. The majority (19/35) preferred hearing the diagnosis early (i.e., before start of symptoms) (Table 1, statement 1). There are multiple arguments that plea for an early diagnosis: uncertainty to diagnosis, early medical access, prenatal diagnostics, and cancer screening for parents (especially breast cancer screening for mothers). In accordance with expectations, many parents experience uncertainty towards the diagnosis A-T (31/35) (Table 1, statement 2). A parent illustrated the time before diagnosis: “We got the diagnosis A-T when our child was eight years old. The time before diagnosis was insecure. We questioned what the future of our daughter would look like. This period was difficult, sad and insecure.” One of the other parents described the effect on their relationship: “The advantage of an early diagnosis to me is: the insecurity is taken away, you can get used to the feeling your child is ill and you can share this feeling with your partner. This leads to better binding between wife and husband. The sharing of grief prevents growing apart.”

**Table 1:** Results of the questionnaire of 35 A-T parents

	Statements	Strongly agree	Agree	Neutral	Disagree	Strongly disagree	Not filled in
1	In retrospect, we preferred hearing the diagnosis A-T (in our case) shortly after birth, eventhough our child did not have symptoms at that time.	43% (15/35)	11% (4/35)	17% (6/35)	14% (5/35)	11% (4/35)	3% (1/35)
2	A diagnosis A-T based on the neonatal bloodspot screening prevents a period of uncertainty (start symptoms to eventual diagnosis). This time was a very uncertain period for me.	49% (17/35)	40% (14/35)	3% (1/35)	9% (3/35)	0% (0/35)	0% (0/35)
3	An early diagnosis gives my child early medical access. My child would have had an advantage to have that access.	51% (18/35)	20% (7/35)	6% (2/35)	20% (7/35)	3% (1/35)	0% (0/35)
4	An early diagnosis offers the opportunity to get access to genetic counseling for a potential child wish. For me, an early diagnosis is important for my future family planning.	51% (18/35)	31% (11/35)	9% (3/35)	6% (2/35)	0% (0/35)	3% (1/35)
5	An early diagnosis means that parents know they are carrier of a mutation in the *ATM* gene and with that an increased risk for cancer. It is an advantage to know this health risk. Therefore, an early diagnosis is important for my child.	37% (13/35)	26% (9/35)	20% (7/35)	9% (3/35)	6% (2/35)	3% (1/35)
6	An early diagnosis of A-T prevents parents from enjoying a healthy baby/child in the first years of its life.	17% (6/35)	26% (9/35)	6% (2/35)	20% (7/35)	31% (11/35)	0% (0/35)

### Medical access to a dedicated A-T referral center

A-T children (and adults) in the Netherlands are seen in our tertiary, national A-T referral center on an annual basis. The support in our center is provided by a large multidisciplinary team of doctors and paramedics. Some of the children receive (prophylactic) antibiotics or temporarily supporting immunoglobulins. However, as stated in the “Introduction” section, no treatment is available yet for A-T. Most parents see a medical advantage for their child with an early diagnosis (25/35) (Table 1, statement 3). Moreover, most parents point out the many diagnostic procedures and its risks towards a diagnosis A-T: “My child had unnecessary diagnostic procedures. We could have taken action on time.” Another parent: “An early diagnosis prevents burdensome or even dangerous diagnostic procedures (e.g., x-rays).” In other words, some parents had the feeling that their child had experienced unnecessary and preventable risks or damage.

### Family planning

Most parents see an early diagnosis of A-T as an important asset for future family planning (29/35) (Table 1, statement 4). For many, it is a decisive argument. Parents comment: “I want to make a conscious decision in my family planning” or “Whenever parents want more children, it is good to know there is a problem early on” or “The diagnosis in my first child took a long time. Meanwhile she got a little sister, who was 6 months old when the diagnosis A-T was made. The insecurity about her little sister was substantial. If I had known earlier, she would have been our only child. The risk is too high for me. Happily, her little sister is healthy.”

### Early screening for family members

The majority of parents see an early diagnosis of A-T as an advantage (21/35) (Table 1, statement 5) in order to be aware of their own health risk (increased risk for cancer). Importantly, in the Netherlands, female *ATM* mutation carriers have an adjusted screening program for breast cancer. A parent comments on this advantage: “It can be vital for an *ATM* mutation carrier to know to be at increased risk for cancer.”

### Happy years

The most important argument against an early diagnosis of A-T is the possible loss of happy years. Here, parents have differing opinions, 16 do agree that an early diagnosis would affect the first years of life and 18 do not (Table 1, statement 6). Parents who enjoyed the first healthy years stated: “I did not want to miss the first years of carefree enjoying my child.” Another parent illustrated the fear that the diagnosis A-T brings: “In our case an early diagnosis would have led to more years of worries and fear. We had seven years without big worries, those are very precious to me.” The ones who disagreed with the concept of happy years bring in the insecurity before diagnosis, illustrated in the earlier paragraph about time to diagnosis. One parent explained: “We had many worries and much insecurity. Our general practitioner and the regional hospital did not listen to us. I think we would enjoy this period more if we would have known the diagnosis.”

### Choice

Parents were asked to make a choice for or against the option for an early diagnosis of A-T in the asymptomatic phase. Twenty-six parents think the advantages outweigh the disadvantages, five parents think the disadvantages outweigh the advantages, and four parents do not know the answer to this question. Those who opposed against an early diagnosis were all parents of classic A-T patients (with differing ages). Afterwards, parents were asked what the decisive argument was in their definitive choice. Many of these arguments were discussed in the previous paragraphs: the proponents brought two main arguments to the fore, namely (1) the uncertainty to diagnosis and its potential harmful diagnostic procedures and (2) the considerations in their family planning. The opponents emphasized the meaning of having a seemingly healthy child and with this the golden year(s).

The last question about the theoretical situation whether a test that could detect all patients with A-T via NBS should be implemented gave a similar result: 25 parents agreed, eight disagreed, and two did not fill in an answer.

## Discussion

After 92 years, since the discovery of A-T, our study seems to be the first study to explore the various concerns and anguishes A-T parents face before diagnosis and the impact of delayed diagnosis by answering a semi-structured questionnaire. NBS for SCID unexpectedly creates an opportunity for a very early A-T diagnosis. The benefits for SCID are clear: preventing severe infections by treatment with an early stem cell transplant, which improves life expectancy in this group [9]. Although this is a major argument to implement NBS for SCID, the benefits for the outcome of A-T patients are less clear. What started as a questionnaire to investigate parents’ opinion about a current discussion in the Netherlands gave us an insight in parents’ experience with A-T. Many parents had experienced uncertainty towards the diagnosis, having the feeling that their child is ill and that medical teams are not recognizing this. In these cases, the diagnosis meant a form of relief. Also, parents experienced that their child had been subjected to unnecessary potentially dangerous procedures, e.g., radiation as patients with A-T have an increased radiosensitivity. On the other hand, some parents who only had mild medical issues with their “clumsy” child had experienced the joy of seeing a (seemingly) healthy child grow up (also known as the “golden year(s)”).

The knowledge of having a child with a genetic disease is important for parents. Many of them are young families with an ongoing child wish; others have a completed family but would have decided otherwise if they knew the diagnosis A-T at that time.

In contrast to our expectations, A-T parents were divided about the loss of golden year(s). We expected that there was at least a loss of potential golden year(s) for all parents (also for those who favored an early diagnosis). In this questionnaire, many parents commented on the insecurity before diagnosis and the relief of the eventual diagnosis. These comments have emphasized the impact of not having a diagnosis. Also, parents confirm the importance that there is an advantage for heterozygous *ATM* carriers as the increased risk for breast cancer requires early screening [20].

In the first statement, 19/35 parents would have preferred a diagnosis shortly after birth and 9/35 parents preferred not to hear the diagnosis at that time. As A-T is a devastating diagnosis that no parent ever wants to hear, it is most likely a negative memory which can influence a parent’s opinion in this question. In the final question, we gave parents the opportunity to make a final choice taking all the advantages and disadvantages in their specific situation into consideration. The answer was based on parents’ personal perspectives as parents could not decide for the entire group. We can imagine that parents would choose differently when they would have to make a decision for the whole group, instead of their specific personal situation, considering the complexity of this dilemma.

So far, limited studies show the percentages of A-T newborns that can be diagnosed as a result of NBS by retrospective analysis of NBS of Guthrie cards. At first, a Californian group investigated the records of an A-T cohort over 25 years. Seven samples had low TREC levels in a cohort of 13 A-T patients (54%) [14]. In a small Swedish study, all four patients showed reduced numbers of TRECs. In the Dutch A-T cohort, we tested five Guthrie cards and four had low TRECs (unpublished data). Altogether, these data suggest that the majority of A-T patients will present with low TREC levels at birth and can possibly be diagnosed with A-T as a result of SCID screening. In the questionnaire presented to the A-T parents, no exact numbers were mentioned as these are limited data.

A-T is a very rare disease with unique features. However, similar dilemmas about NBS have been discussed for other diseases, such as Duchenne muscular dystrophy (DMD) and spinal muscular atrophy (SMA). For these disorders, studies similar to the present one that we have performed for A-T showed a strong wish of parents (majority up to 95.9%) to implement DMD and SMA in neonatal screening programs without any therapeutic consequences at the time of the study [22]. In the 1980s, when the screening for DMD was first discussed, a similar retrospective study was performed to objectify parents’ opinion on neonatal screening: a similar percentage of parents (75%) was in favor of an early diagnosis, based on the same arguments as diagnostic delay, practical advantages, family planning, and emotional advantages [7]. In semi-structured interviews, parents reflected on the (delayed) diagnostic process and emphasized their feelings of worry and anxiousness of having an undiagnosed ill child and the eventual relief of being guided by a dedicated DMD team [3]. In all these aspects, our study shows similarities to the studies about screening for this untreatable disorder.

At this moment, a curative treatment for A-T is not available. NBS may identify pre-symptomatic patients, while—on the contrary—recent studies have also identified patients at the highest risk for early morbidity and mortality [21]. Whenever a form of treatment becomes available, these groups at both ends of the clinical spectrum may be the first to benefit from early medical intervention. Undoubtedly, new technologic developments will influence the discussion about NBS for A-T.

This study has some limitations: it is a relatively short questionnaire in a small cohort. With only little number of cases, no subgroup analysis was possible. Despite the limitations, however, we feel it is valuable to share the opinion of our cohort of A-T parents. Future research should address structured interviews with A-T parents. In addition, non-A-T parents should be introduced in this subject and asked for their opinion as well. This way, the current health policy regarding SCID screening in the Netherlands could be re-evaluated taking (A-T) parents’ perspectives into consideration.

## Conclusion

The majority of parents of A-T patients would prefer to know the diagnosis A-T shortly after birth of their child, based on two major arguments: (1) the experienced insecurity in diagnostic trajectories and its impact on their lives and (2) the knowledge of being *ATM* mutation carriers when making decisions about family planning. Parents who opposed against an early diagnosis emphasized the joy of having a seemingly healthy child until diagnosis.

## Electronic supplementary material

ESM 1 (PDF 288 kb)

## Abbreviations

A-T: Ataxia telangiectasia
ATM: Ataxia Telangiectasia Mutated (gene)
DMD: Duchenne muscular dystrophy
METC: Medical ethical committee
NBS: Newborn screening
SCID: Severe combined immunodeficiency
SMA: Spinal muscular atrophy
SONNET(-study): SCID-screening Research in the Netherlands with TRECs
TREC: T cell receptor excision circle
