# Supplementary material for: Early diagnosis of ataxia telangiectasia in the neonatal phase: a parents’ perspective
Source: Eur J Pediatr. 2019 Nov 11;179(2):251–6. doi: 10.1007/s00431-019-03479-5 (PMC6970962; doi:10.1007/s00431-019-03479-5)
Supplement: Supplementary file 1 — (PDF 288 kb) [file 431_2019_3479_MOESM1_ESM.pdf]

Questionnaire for parents of a child with Ataxia Telangiectasia (A-T)

Newborns in the Netherlands are tested for a number of rare, serious and treatable diseases via the newborn screening program. Timely detection and treatment of these diseases will prevent or limit serious health damage. In 2017, the State Secretary for Health, Welfare and Sport decided to expand the Dutch newborn screening program with twelve new diseases. One of these new diseases is Severe Combined Immunodeficiency (SCID), a rare, serious disease of the immune system that can be cured with stem cell transplantation. In the newborn screening test for SCID, certain markers of the immune system are low or absent at birth. These markers can also be low or absent in A-T patients. As a result, children with A-T may have an abnormal newborn screening result, even though there are no symptoms of A-T in the neonatal period. We wonder what the advantages and disadvantages are of an early diagnosis A-T. We believe that the experience and opinion of A-T parents are important aspects in this discussion. We would therefore like to present a number of statements to gain more insight into the considerations regarding an early diagnosis of A-T, from the perspective of parents.

Father’s name:

Mother’s name:

Name and date of birth child:

We would prefer it if both parents filled in a questionnaire separately.

This form was filled in by:

Father   Mother   Father and mother together

Statements

After each statement, you can choose between five possibilities: strongly agree – agree- neutral – disagree – strongly disagree. Please encircle the answer that best applies to you.

Statement. In retrospect, we would rather have heard the diagnosis A-T of our child shortly after birth, even though our child had no symptoms of the disease at that time.

Your opinion:

1. Strongly agree. 2. Agree. 3. Neutral. 4. Disagree. 5. Strongly disagree.

Various considerations could play a role in the answer to the above question. We would like to know your opinion about early diagnostics (shortly after birth). Could you therefore indicate to what extent you agree with the following statements?

Statement. A diagnosis A-T based on newborn screening prevents a period of uncertainty (from start of symptoms to final diagnosis).

Your opinion:

There was a time where symptoms of A-T were present, but there was no diagnosis yet. This was a very uncertain period for me.

1. Strongly agree. 2. Agree. 3. Neutral. 4. Disagree. 5. Strongly disagree.

Statement. An early diagnosis of A-T gives my child early medical access. Therefore, supportive therapy can be started in an early phase of the disease.

Your opinion:

My child would have had an advantage to have that early medical access.

1. Strongly agree. 2. Agree. 3. Neutral. 4. Disagree. 5. Strongly disagree.

Statement. An early diagnosis of A-T offers the opportunity to get access to genetic counselling.

Your opinion:

For me, an early diagnosis is important for my future family planning.

1. Strongly agree. 2. Agree. 3. Neutral. 4. Disagree. 5. Strongly disagree.

Statement. An early diagnosis of A-T deprives parents of the opportunity to enjoy a (seemingly) healthy newborn/child in the first years of life.

Your opinion:

I would have enjoyed my child's early childhood less if I had known the diagnosis A-T shortly after birth.

1. Strongly agree. 2. Agree. 3. Neutral. 4. Disagree. 5. Strongly disagree.

Statement. An early diagnosis of A-T means that parents will also know that they are carriers of a mutation in the *ATM* gene with an (mildly) increased risk of developing cancer.

Your opinion:

It is an advantage to be aware of the above-mentioned health risk for parents (and other family members). That is why an early diagnosis is important for both me and my child.

Space for any additional comments or suggestions:

Do you have arguments to be for or against an early diagnosis of A-T, based on newborn screening in the first week of life, other than described in the above statements? If yes, please fill in below:

**For an early A-T diagnosis**	**Against an early A-T diagnosis**

Choice

After you have considered all the above questions and statements, we would like to ask you what you think about the introduction of a new technique in the newborn screening program that leads to an early diagnosis of A-T (in the first weeks of life) in newborns in the Netherlands.

The following would apply to me:

1. I am FOR an early diagnosis: the benefits outweigh the disadvantages.
2. I am AGAINST an early diagnosis: the disadvantages outweigh the benefits.
3. I don’t know

**What is the decisive argument in your consideration?**

Other questions

Finally, we have two statements to gain insight into the possible follow-up procedure that we should follow with abnormal screening results.

Statement. With the current technique, A-T is "accidentally" detected as an incidental finding of newborn screening for SCID. However, the screening is intended to diagnose SCID (see above). In the case of an abnormal SCID screening result that turns out not be SCID after follow-up diagnostics, the newborn could have A-T. In this case, a medical team should keep a close eye on the patient, but diagnostics for A-T should not be applied. Additional diagnostics for A-T should only be used if symptoms of A-T begin to occur

Your opinion:

1. Strongly agree. 2. Agree. 3.Neutral. 4. Disagree. 5. Strongly disagree.

Statement. If another technique was available that would be able detect all children with A-T with newborn screening, A-T should be included in the regular Dutch newborn screening program.

Your opinion:

1. Agree, the benefits outweigh the disadvantages.
2. Disagree, the disadvantages outweigh the benefits.

Thank you for completing this questionnaire.

Space for any additional comments or suggestions:
